# mRNA Biomarkers in Dried Blood Spots May Improve Detection of Autologous Blood Micro‐Transfusions Using an Individualized Approach

**DOI:** 10.1002/dta.3939

**Published:** 2025-08-09

**Authors:** Andreas Breenfeldt Andersen, Jessica Almeida Oliveira, Francesco Loria, Jacob Bejder, Olivier Salamin, Tiia Kuuranne, Nikolai B. Nordsborg, Nicolas Leuenberger

**Affiliations:** ^1^ Department of Public Health, Research Unit for Exercise Biology Aarhus University Aarhus Denmark; ^2^ Swiss Laboratory for Doping Analyses, University Center of Legal Medicine, Lausanne & Geneva Lausanne University Hospital & University of Lausanne Lausanne Switzerland; ^3^ Department of Nutrition, Exercise and Sports (NEXS) University of Copenhagen Copenhagen Denmark

## Abstract

Autologous blood transfusions (ABTs) are prohibited by the World Anti‐Doping Agency (WADA), yet detecting autologous blood micro‐transfusions (ABMTs) remains a challenge. Due to smaller transfused volumes, ABMTs cause attenuated biomarker changes, limiting detection sensitivity within the Athlete Biological Passport (ABP). This study assessed whether mRNA expression of 5‐aminolevulinic acid synthase (*ALAS2*) and carbonic anhydrase 1 (*CA1*), measured from dried blood spots (DBS), could serve as sensitive biomarkers of ABMT. In a randomized, placebo‐controlled design, 47 trained individuals (24 ♀; mean VO_2_peak 56 ± 7 mL·min^−1^·kg^−1^) were allocated to an ABMT group (*n* = 23; ♀ = 12) or placebo group (*n* = 24; ♀ = 12). The ABMT group donated 450 mL of blood and received a 130 mL packed red blood cell reinfusion 4 weeks later. Blood sampling occurred regularly before and after both donation and reinfusion. *ALAS2* and *CA1* mRNA expression from DBS, and reticulocyte percentage (RET%) from venous blood, were analyzed. Following blood donation, *ALAS2*, *CA1*, and RET% increased by 270%, 200%, and 150%, respectively. However, no consistent group‐level changes were observed after ABMT. Individualized analysis identified more outliers for *ALAS2* than for *CA1*, and blinded interpretation of individual mRNA profiles achieved > 95% sensitivity and specificity for detecting ABMT. These findings suggest that *ALAS2* mRNA expression, assessed via minimally invasive DBS sampling, is a promising biomarker for identifying ABMT. This approach may enhance current anti‐doping strategies by improving sensitivity to small‐volume autologous transfusions that evade detection through traditional ABP biomarkers.

## Graphical abstract

This study shows that measuring ALAS2 mRNA expression from dried blood spots (DBS) can sensitively identify autologous blood micro‐transfusions (ABMTs) ABMTs with > 95% sensitivity and specificity through individualized analysis. These results highlight ALAS2 mRNA in DBS as a promising tool to strengthen anti‐doping efforts against small‐volume autologous transfusions.

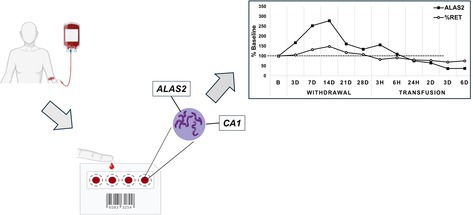

## 1 Introduction

Autologous blood transfusion, that is, withdrawing and later reinfusing the donor's own blood, is prohibited by the World Anti‐Doping Agency (WADA) [1] due to the method's capability to increase aerobic endurance performance and maximal oxygen consumption [2, 3, 4, 5, 6, 7]. However, the detection of autologous blood transfusion remains a challenge. Currently, the prohibited method can only be detected indirectly via the hematological module of the Athlete Biological Passport (ABP) [8, 9, 10] although sensitivity seems to be improved by including other indicators to target testing, such as plasticizers originating from the blood bags and being detected in urine samples [11, 12, 13]. The ABP is based on statistical principles where hematological values are longitudinally compared with the athlete's own historical values that have established the individualized tolerance limits that are capable of flagging atypical values [14, 15]. Specifically, hemoglobin concentration ([HGB]) and the *OFF‐Score* ([HGB] − 60 × √reticulocyte percentage (RET%)) are primary markers in the hematological ABP generating atypical passport findings (ATPF) [16], whereas RET% and a multifactorial composite score, the Abnormal Blood Profile Score (ABPS), are regarded as secondary markers used to complement the primary markers [17]. Importantly, the ABP has previously detected 63% of the individuals donating 450 mL blood [3]. Likewise, when transfusing 450–1350 mL blood in well‐trained cyclists, 20% of the individuals were detected by the ABP, which increased to 80% when using a blinded expert investigator throughout a season [18].

The ABP detection of ABT is further challenged by the possibility that *micro‐dosin*, that is, smaller transfusion volumes defined as < 450 mL whole blood [4] is being applied as doping practice [19]. We recently demonstrated that as little as ~130 mL packed red blood cells are sufficient to increase time‐trial performance in well‐trained individuals [2, 3]. Here, the ABP sensitivity was only 29% with 99% specificity up to 6 days after reinfusion by the ABP [3]. Accordingly, the development of novel biomarkers sensitive to micro‐dosing is warranted.

We have previously demonstrated that two erythropoiesis‐related mRNA biomarkers are potential novel biomarkers for blood transfusion. The expression of 5′‐aminolevulinate synthase 2 (*ALAS2*), a catalyst of the first step in heme biosynthesis (succinyl‐CoA and glycine ➔ 5‐aminolevulinic acid (ALA)) [20] as well as carbonic anhydrase 1 (*CA1*), the key enzyme for regulation of cellular pH (CO_2_ + H_2_O ⇌ HCO_3_^−^ + H^+^) [21] are altered in healthy males following a standard volume blood donation and transfusion [22, 23]. Both mRNA biomarkers were downregulated following an autologous blood transfusion of ~280 mL packed red blood cells, with nadir occurring after 9 days [23, 24]. However, whether the biomarkers are sensitive to micro‐dosing of red blood cells remains unknown.

One major advantage of the mRNA biomarkers is the option of reliably quantifying their level in dried blood spots (DBS) [22]. The collection of DBS has numerous advantages compared with the traditional venous blood sampling. For instance, the collection is less invasive, the sample has higher stability for mRNA at room temperature, and can be shipped with no time or temperature restrictions, which can reduce sample shipping costs significantly [22, 25, 26]. According to feedback from the field, DBS is also preferred over venous blood collection by athletes and doping control officers [27] and has successfully been applied for routine anti‐doping analyses [28, 29]; [30].

To tackle the analytical challenges of ABMT, the aim of the present study was to investigate the hypothesis that *ALAS2* and *CA1* mRNA expression are sensitive and specific biomarkers for the identification of phlebotomized and micro‐transfused individuals using DBS as a sample matrix.

## 2 Materials and Methods

### 2.1 Participants and Study Design

**FIGURE 1:**
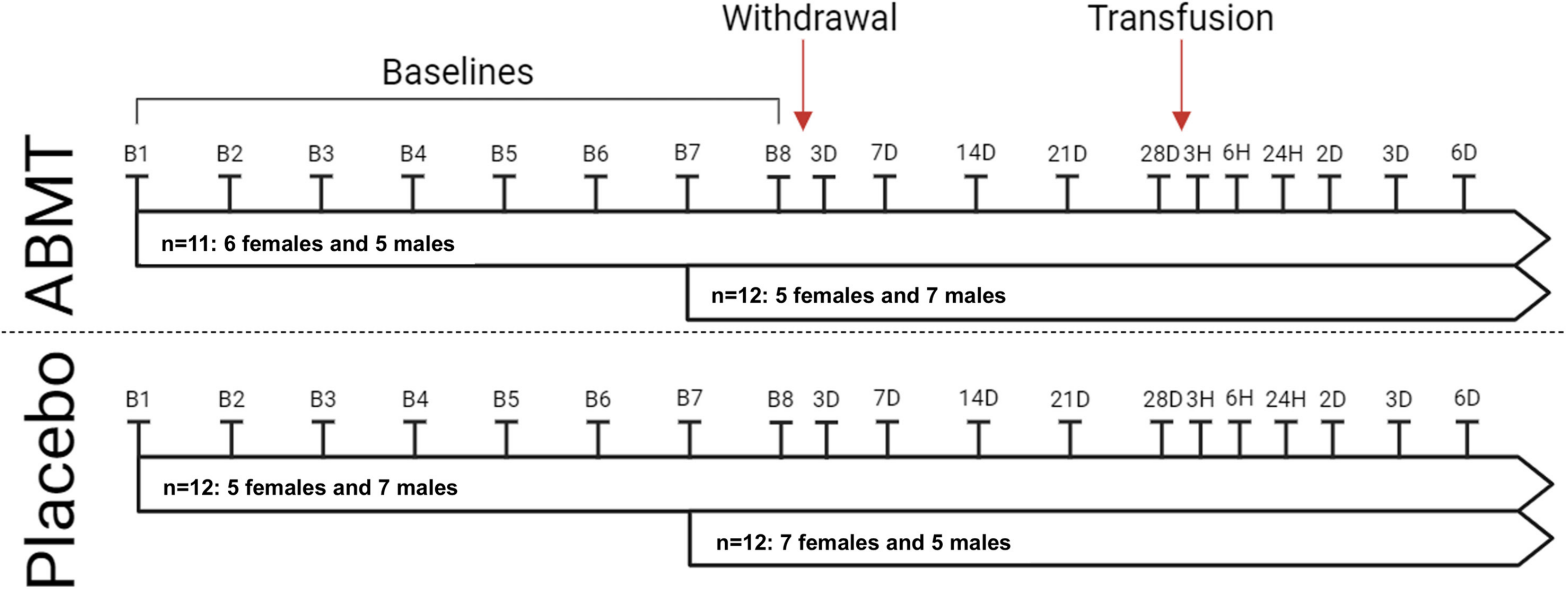
Study design. Autologous blood micro‐transfusion (ABMT). B1–8: Baseline number; *#*D: Number of days after either donation or transfusion; #H: Number of hours after transfusion.

The study was approved by the ethics committee of Copenhagen University (Copenhagen, Denmark; H‐17024876) and was performed in accordance with the Declaration of Helsinki. The study design and participant characteristics have been described in detail elsewhere [3]. Briefly, 47 trained and healthy males (*n* = 23) and females (*n* = 24), all living at sea level and who had not been exposed to high altitude or donated blood in the last 3 months prior to the study, gave informed consent to the present analyses. Following inclusion, the participants were randomized to either an autologous blood micro‐transfusion (ABMT; *n* = 23) or placebo group (*n* = 24). Participants were further randomized into a long (8 weeks; *n* = 24) and short baseline period (2 weeks; *n* = 23) with DBS samples collected weekly (Figure 1). After the baseline period, the ABMT group donated 450 mL whole blood and the fractionated red blood cells were stored at 4°C for 4 weeks. DBS was collected by spotting capillary blood directly from the top of the fingers on a filter paper (Whatman FTA DMPK‐C, Qiagen, Hilden, Germany) at days 3, 7, 14, 21, and 28 after donation. Four weeks after the donation, participants in the ABMT group were transfused with 128 ± 6 mL packed RBC obtained from the phlebotomy, followed by collection of DBS after 3, 6, and 24 h, and 2, 3, and 6 days. Samples were collected from the placebo group at the same time points, but participants in the placebo group did not undergo any phlebotomy or transfusion [3].

### 2.2 Erythropoiesis‐Related mRNA Extraction and Analysis

Extraction of erythropoiesis‐related mRNA and RT‐qPCR analysis were performed as described previously [31, 32, 33]. After the extraction, RT‐qPCR analysis was performed as it has been performed in previous studies [31, 32, 33]. Three housekeeping genes (GAPDH, RGCC L, and RGCC C) were used to normalize the results, allowing the use of DBS with different volumes. The hematological results from venous blood analysis, including RET%, have been published previously [3]. Here, we included RET% biomarker since previous studies have described its applicability as an appropriate comparator for *ALAS2* and *CA1* mRNA expression levels [22, 31].

### 2.3 Intra‐Individual Variability Calculation

Intra‐individual variability was assessed using the percentage coefficient of variation (%CV) in 12 females and 12 males from the placebo group, as described in Salamin et al. [34]. Briefly, the mean and standard deviation (SD) of the measured values for *ALAS2*, *CA1*, and RET% were calculated for each participant across all time points. The %CV was then obtained by dividing the SD by the corresponding mean value.

### 2.4 Passport Approach

To mimic the actual hematological ABP with the most appropriate method, a passport simulation, using an athlete passport approach for *ALAS2* and *CA1* analysis based on WADA guidelines [30], was completed for each participant in both groups as described previously [31]. The population mean (POP_mean_) mRNA expressions relative to housekeeping genes and between‐subject variance (BS_var_) were calculated using all baseline points for all participants and were POP_mean_, *ALAS2* = 7.51 and *CA1* = 2.21, and BS_var_ were *ALAS2* = 18.68 and *CA1* = 0.84). Within‐subject variation (WS_var_) was calculated independently for each participant using their baseline data, which consisted of either two or eight samples as described in the study design. The WS_var_ values (mean ± standard deviation (SD)) were *ALAS2* = 5.39 ± 7.51, *CA1* = 0.43 ± 0.99.

### 2.5 Blinded Analysis

A non‐biased interpretation of the investigated biomarkers *ALAS2*, *CA1*, and RET% data were completed by two blinded investigators without prior knowledge of group allocation. The blinded investigators were asked to conclude whether a participant was part of the ABMT or placebo group based on the entire time series. Each interpretation was performed using all the collected samples from the individual to allow for a high time resolution.

### 2.6 Statistics

To investigate the effect of blood withdrawal and reinfusion on *ALAS2* and *CA1* mRNA expression as well as RET%, a linear mixed‐effects model was used. Prior to modeling, the data were log‐transformed to ensure normality, as visual inspection by QQ‐plots and the Shapiro–Wilk test indicated a non‐normal distribution in the raw data. Baseline measurements were combined for the statistical analysis to represent a unified baseline condition, as subsequent analyses focused on the percentage of change relative to baseline (baseline set to 100%). The model included “Time” and “Group” as fixed effects, along with their interactions, while including “Participants” as a random effect to account for inter‐subject variability. This model allowed the evaluation of the main effects of time, group, and their interactions while accounting for repeated measurements within subjects. Missing values were handled using the *na.exclude* method to ensure that incomplete observations did not bias the results. Estimated marginal means were calculated for each combination of time and group using the *emmeans* package in R [35]. Post hoc pairwise comparisons were conducted to assess within‐group differences across time points and between‐group differences at each time point. These comparisons were adjusted for multiple testing using the Bonferroni correction, with a significance threshold of *p* < 0.05.

Unless stated otherwise, figures illustrate the percentage of change in *ALAS2*, *CA1* levels, and RET% relative to baseline. All analyses were performed using R Studio, with statistical significance assessed at a two‐tailed alpha level of 0.05.

For calculation of inter‐individual variability, unpaired t‐tests were used to assess the statistical significance for %CV comparison between *ALAS2*, *CA1*, and RET% as described previously [36].

For the simulated passport approach, the number of samples exceeding the calculated thresholds within the ABMT group (i.e., true positives), and the number of samples exceeding the calculated thresholds within the placebo group (i.e., false positives) were used to determine the sensitivity and specificity, respectively, for each marker alone (*ALAS2* and *CA1*). For the blinded interpretation, specificity was calculated by dividing true negative interpretations by all true negative individuals; sensitivity was calculated by dividing true positive interpretations by all true positive individuals.

## 3 Results

**FIGURE 2:**
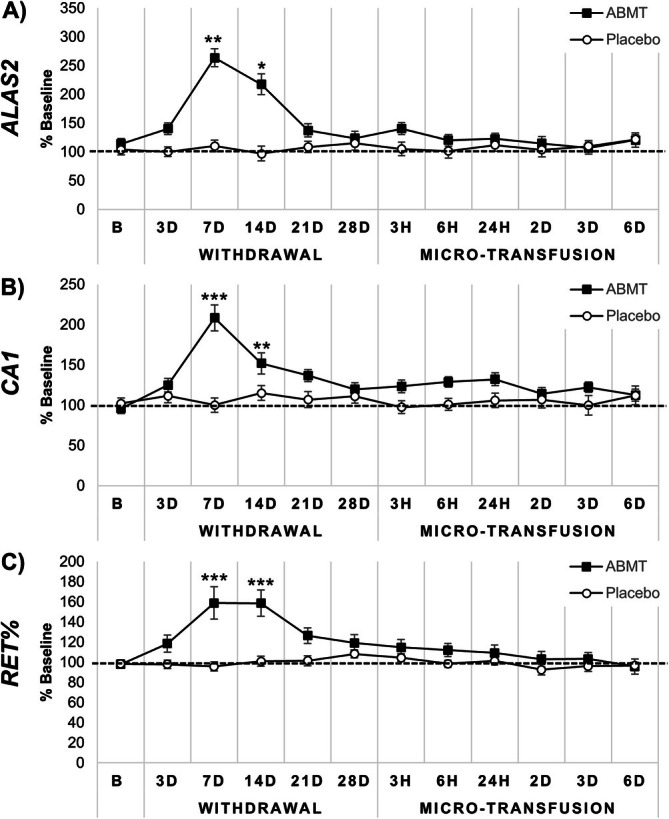
Comparison of (A) *ALAS2*, (B) *CA1*, and (C) RET% biomarkers in the autologous blood micro‐transfusion (ABMT; ■, *n* = 23) and placebo (○, *n* = 24) groups. Data are presented as percentages of baseline values (y‐axis). “B” indicates the mean of baseline sample values (two baseline samples, *n* = 24; eight baseline samples *n* = 47), “3D–28D” indicates 3–28 days after blood withdrawal, “3H, 6H, and 24H” indicates “3, 6, and 24H” after micro‐transfusion, and “2D, 3D, and 6D” indicate 2, 3, and 6 days after micro‐transfusion, respectively. Significant values regarding between‐group and within‐group changes related to baseline are marked with an asterisk (*) for *p* ≤ 0.05, ** for *p* ≤ 0.01 and *** for *p* ≤ 0.001.

In general, both *ALAS2*, *CA1*, and RET% displayed similar profiles, characterized by a clear increase after blood withdrawal (Figure 2). A significant time x group effect (*p* < 0.01) was observed for RET%, *ALAS2*, and *CA1*. In the autologous blood micro‐transfusion (ABMT) group, all three biomarkers increased significantly 7 and 14 days after the 450 mL blood donation compared with baseline and the placebo group (Figure 2A–C). The greatest fold‐change observed occurred after 7 days for *ALAS2*, *CA1*, and RET% (2.7 ± 0.8‐fold, *p* < 0.001; 2.0 ± 0.8‐fold, *p* < 0.001; 1.5 ± 0.3‐fold, *p* < 0.001 respectively; Figure 2A–C). Following ABMT of 130 mL packed red blood cells, no between‐ or within‐group changes were found.

**FIGURE 3:**
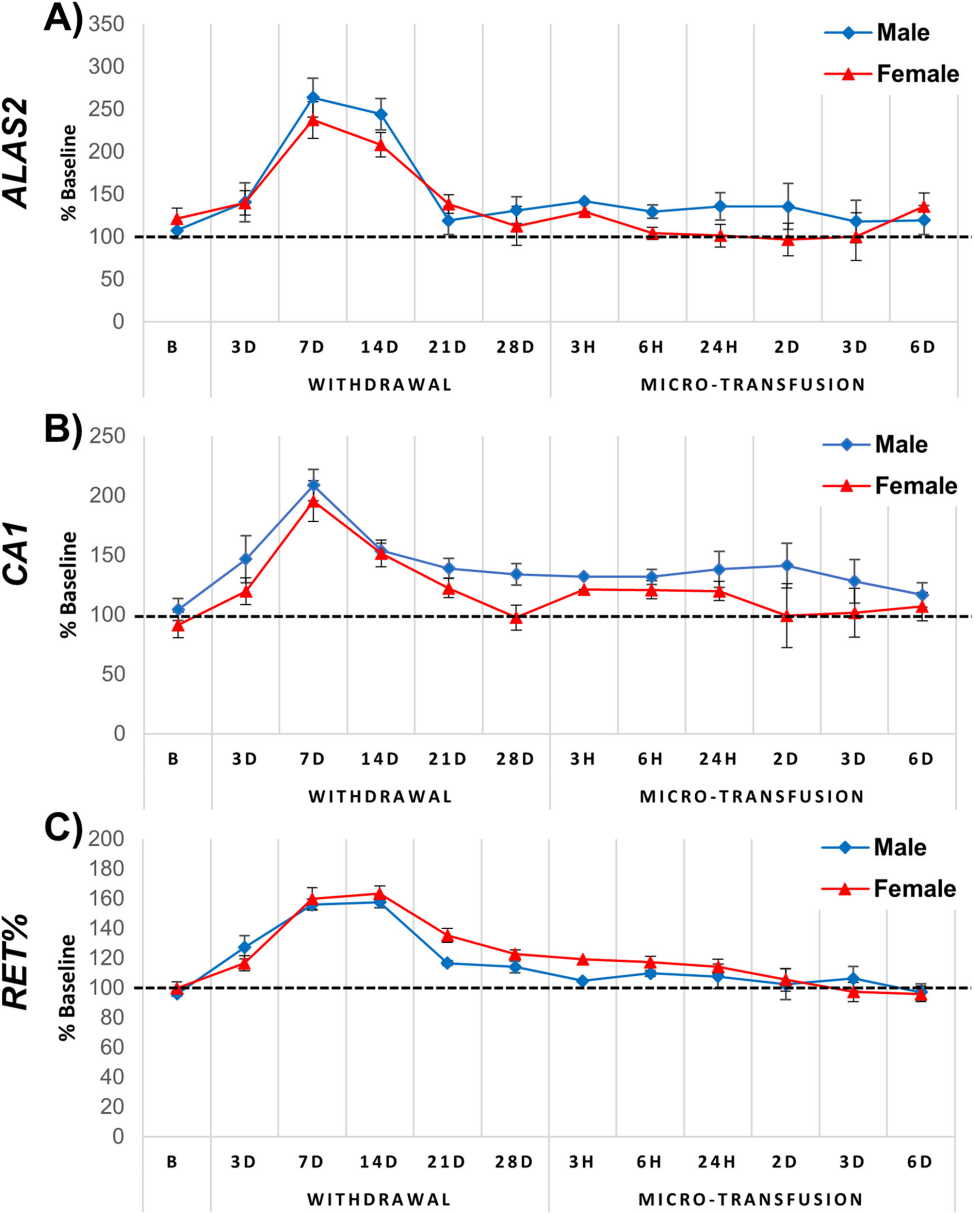
Comparison of (A) *ALAS2*, (B) *CA1*, and (C) RET% in male (⬥ blue, *n* = 23) and females (▲ red, *n* = 24) in the ABMT group. Data are presented as percentages of baseline values (*y*‐axis). “B” indicates the mean of baseline sample values (two baseline samples, *n* = 24; eight baseline samples *n* = 47), “3D–28D” indicates 3–28 days after blood withdrawal, “3H, 6H, and 24H” indicates “3, 6, and 24H” after micro‐transfusion, and “2D, 3D, and 6D” indicate 2, 3, and 6 days after micro‐transfusion, respectively.

**FIGURE 4:**
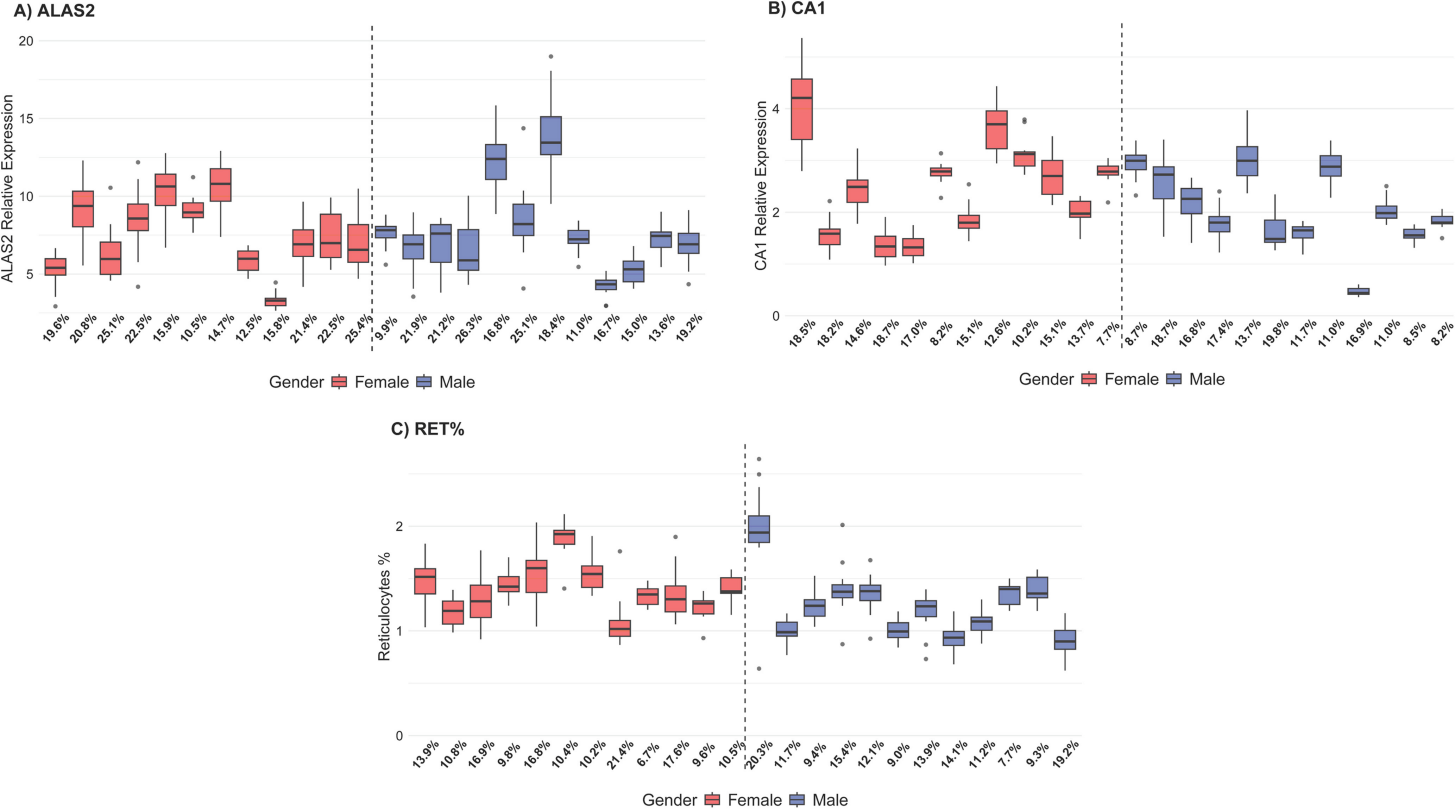
Intra‐individual variability. Longitudinal monitoring of (A) *ALAS2*, (B) *CA1*, and (C) RET% levels in participants from the placebo group. *x*‐axis represents coefficient of variation percentage (CV%).

No sex‐specific differences were observed when comparing the changes in *ALAS2*, *CA1*, and RET% in males and females (Figure 3). The average mRNA expression level relative to housekeeping genes of *ALAS2* was 7.4 ± 2.2 (3.3 to 10.5) in females and 7.8 ± 2.7 (4.3 to 13.9) in males, with intra‐individual variability ranging from 10.5% to 25.4% in females and 9.9% to 26.3% in males (Figure 4A). For *CA1*, the average relative expression level was 2.5 ± 0.9 (range 1.4 to 4.0) in females and 2.1 ± 0.7 (range 0.5 to 3.0) in males (Figure 4B), with intra‐individual variability calculated at 7.7%–18.7% for females and 8.2%–19.8% for males. The average RET% level was 1.40 ± 0.21% (range from 1.19% to 1.88%) in females and 1.24 ± 0.31% (0.89% to 2.04% in males, with intra‐individual variability ranging from 6.7% to 21.4% in females and 7.7% to 20.3% in males (Figure 4C). The mean intra‐individual variability of *CA1* and RET%, irrespective of sex, was significantly lower when compared with *ALAS2* (18.4 ± 4.9% for *ALAS2* vs. 13.8 ± 4.0% for *CA1*, *p* < 0.001 and 12.8 ± 3.8% for RET%, *p* < 0.0001). No difference was observed when comparing the mean intra‐individual variability of *CA1* and RET%.

**FIGURE 5:**
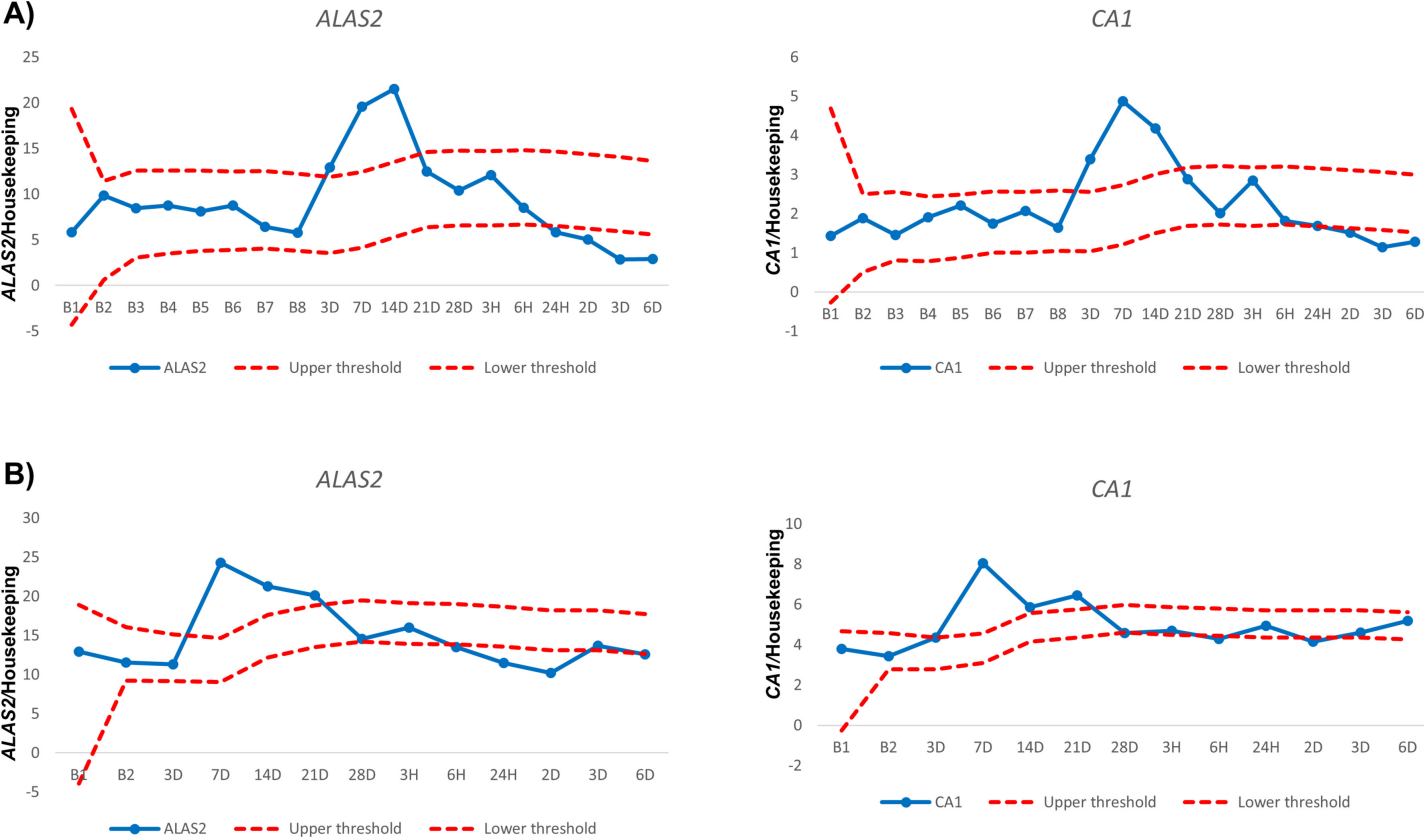
Hematological passport‐like approach for two ABMT treated subjects (A) and (B) for *ALAS2* and *CA1*. Red lines indicate upper and lower thresholds calculated with an athlete biological passport‐like approach.

**FIGURE 6:**
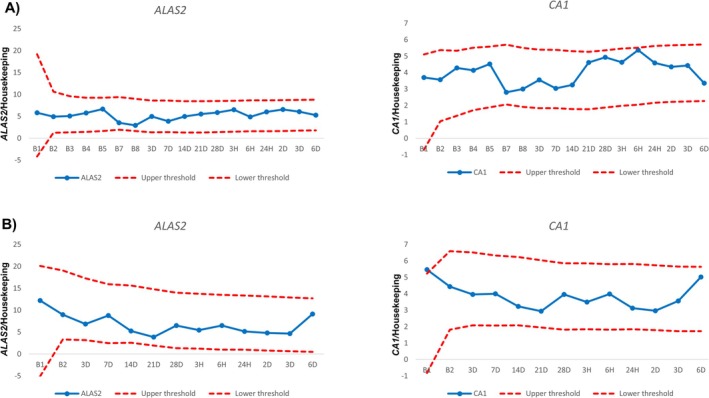
Hematological passport‐like approach for two placebo subjects (A) and (B) for *ALAS2* and *CA1*. Red lines indicate upper and lower thresholds calculated with an athlete biological passport‐like approach.

**FIGURE 7:**
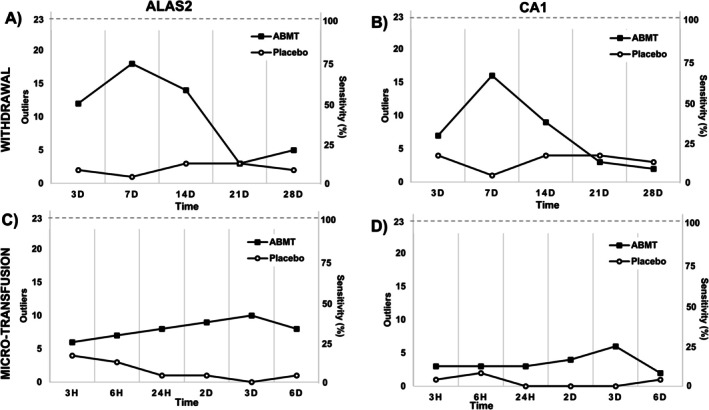
The abilities of the RNA biomarkers to detect withdrawal and micro‐transfusion. Number of outliers in the autologous blood micro‐transfusion (ABMT) and placebo groups after withdrawal and micro‐transfusion for each volunteer at each time point for all biomarkers. Black squares, ABMT group. White circles, placebo group. The x‐axis represents sample time points. The y‐axis indicates the number of volunteers that were flagged (total, *n* = 23). (A) Withdrawal ALAS2 outliers. (B) Withdrawal CA1 outliers. (C) Micro‐transfusion ALAS2 outliers. (D) Micro‐transfusion CA1 outliers.

**FIGURE 8:**
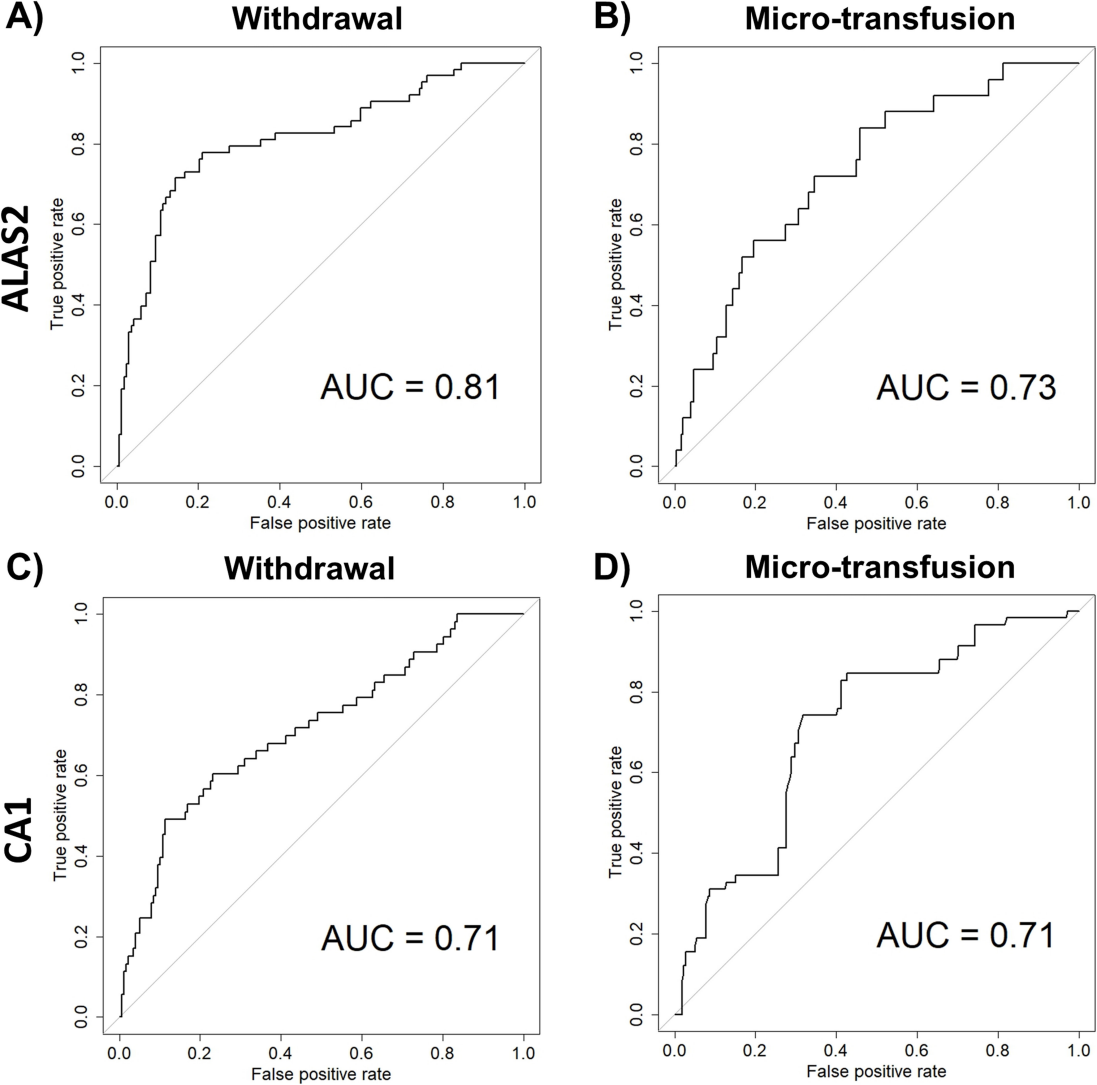
Receiver operating characteristic curves. True and false positive rate for ALAS2 (A) and (B) and for CA1 (C) and (D) in withdrawal and micr*o*‐transfusion times points, respectively.

When applying an ABP‐like approach, the number of outliers from all participants was accumulated and plotted according to time point as exemplified in Figure 5 (ABMT), Figure 6 (Placebo) and in Table S1 (see method in [31]). If a sample crossed either the upper or lower threshold, it was defined as an outlier. *ALAS2* had numerically more outliers at all time points after withdrawal and reinfusion when compared with *CA1* (Figure 7A–D). Most outliers occurred between 3 and 14 days after blood withdrawal (Figure 7A,B) with a peak for *ALAS2* and *CA1* of 18 and 16 outliers, respectively. Following the micro‐transfusion, *ALAS2* also displayed numerically more outliers compared with *CA1* (Figure 7C,D). The number of outliers in the ABMT group showed a linear increase from 3 h to 3 days after micro‐transfusion, followed by a decrease after 6 days. The number of *CA1* outliers remained slightly higher than those for the placebo group, with a small increase observed 3 days after micro‐transfusion. For the simulated passport approach, the number of samples exceeding the calculated thresholds within the ABMT group (i.e., true positives) and the number of samples exceeding the calculated thresholds within the placebo group (i.e., false positives) was used to determine the sensitivity and specificity, respectively, for each marker (*ALAS2* and *CA1*) (Table 1). An overview of specificity and sensitivity showed that *ALAS2* had an area under the curve (AUC) of 0.81 and 0.73 following blood withdrawal and ABMT, respectively. *CA1* had an AUC of 0.71 and 0.71 with withdrawal and micro‐transfusion (Figure 8).

**TABLE 1:** Specificity and sensitivity results in withdrawal and transfusion phases for *ALAS2* and *CA1*.

	Withdrawal	Transfusion
Specificity		
*ALAS2*	97.7%	91.7%
*CA1*	87.6%	96.7%
Sensitivity		
*ALAS2*	65.2%	41.7%
*CA1*	33.6%	18%

The blinded interpretation of all time‐points for each individual resulted in correct classification of 23 out of 24 true negative individuals as well as 22 out of 23 true positive individuals, providing a specificity of 95.8% and a sensitivity of 95.7%.

**FIGURE 9:**
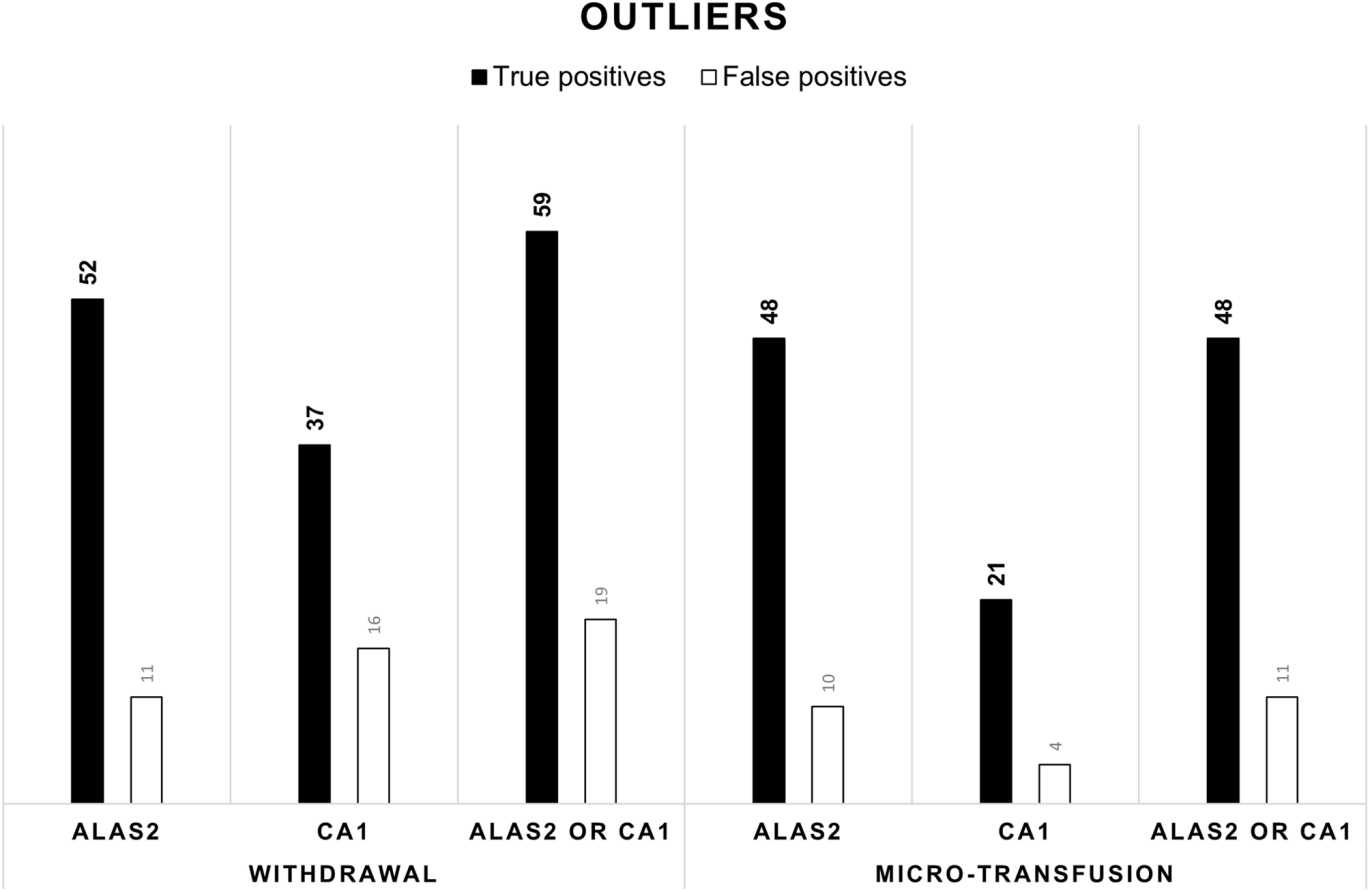
Sum of outliers in the autologous blood micro‐transfusion (ABMT; black) and placebo groups (white) based on withdrawal (*n* = 115) and transfusion (*n* = 138) samples for each biomarker and their combination. Black bars indicate true positives. White bars indicate false positives.

An investigation was conducted to determine whether the outliers identified by *CA1* coincided with those detected by *ALAS2*. The total numbers of outliers associated with true and false positives in the ABMT and placebo subgroups for withdrawal and micro‐transfusion are summarized as a histogram in Figure 9. For withdrawal, *ALAS2* resulted in a numerically higher number of true positives and a lower number of false positives than those for *CA1*. For micro‐transfusion, both true and false positives were numerically higher for *ALAS2* than for *CA1*.

## 4 Discussion

This is the first study evaluating the impact of a standard blood donation and subsequent ABMT on the mRNA biomarkers, *ALAS2* and *CA1* in DBS samples. We investigated if they could serve as supplementary markers to the ABP using RET% as a reference marker. *ALAS2*, *CA1*, and RET% showed similar profiles, characterized by mean increases following blood withdrawal (Figure 2) of 2.7 ± 0.8‐fold, 2.0 ± 0.8‐fold, and 1.5 ± 0.3‐fold, respectively. Following the transfusion of 130 mL, no significant changes were found. Analyzing the entire study period (withdrawal and transfusion) using a passport approach revealed more outliers in general using *ALAS2* than with *CA1*, and the majority of *CA1* outliers were also detected using *ALAS2*.

Our findings corroborate previous reports investigating the promising potential of *ALAS2* and *CA1* for detecting illicit erythropoietic manipulations. Both *ALAS2* and *CA1* have been proven sensitive as markers for ABT with larger volumes [23, 24] and administration of micro‐doses of EPO [31, 37]. Our team has previously demonstrated that rHuEPO affects the levels of *ALAS2* and *CA1* and that these biomarkers are also influenced by altitude, although less than rHuEPO [31]. We concluded that *ALAS2* and *CA1* biomarker levels increase with rHuEPO micro‐doses, in a similar manner to RET% but with a higher amplitude [31]. Despite clear changes observed following blood donation, where the *ALAS2* and *CA1* response was more marked (2.7 ± 0.8‐fold and 2.0 ± 0.8‐fold, respectively; Figure 2A,B) than that of RET% (1.5 ± 0.3‐fold; Figure 2C), no changes in mean values were found following the ABMT (Figure 2). Previously, the maximal changes in *ALAS2* and *CA1* following a 280 mL blood transfusion have been reported to occur after 9 days [23] while the last sample in the present study was collected 6 days after the reinfusion. It is likely that the follow‐up period of 6 days did not allow us to observe the full response in the investigated biomarkers. As the investigated markers and RET% are closely related, this may be partially explained by the reticulocyte maturation process lasting 6–10 days [38].

The signal‐to‐noise ratio is crucial for potential new biomarkers serving as indirect cues of blood manipulation. Thus, for *ALAS2* and *CA1* to complement the ABP appropriately, a high degree of sensitivity and specificity is required. The intra‐individual variability of *ALAS2* and *CA1* levels was found to range between 10.5%–25.4% and 7.7%–18.7% for females, respectively, and 9.9%–26.3% and 8.2%–19.8% for males, respectively (Figure 4). In comparison, RET% variability ranged from 6.7%–21.4% and 7.7%–20.3% for females and males, respectively, which is lower than the variability observed for *ALAS2* expression in this study (Figure 4). However, the responses of *ALAS2* to blood manipulation are more pronounced than those of RET%, as demonstrated in Figure 2 and corroborated by previous studies [22, 23, 31]. Interestingly, the mean intra‐individual variability of *CA1* (13.83%) is comparable to that of RET% (12.80%) and *CA1* exhibits more marked responses to blood manipulation than RET% (Figure 2). Importantly, the variability of *ALAS2* and *CA1* was of a similar magnitude as previously observed among currently implemented serum markers in the steroidal module of the ABP [36]. Thus, considering the variability of *ALAS2* and *CA1*, our findings emphasize their potential utility as complementary biomarkers in the hematological module of the ABP.

Since no WADA monitoring program is yet available for these biomarkers, we applied a passport‐like approach to reveal individual atypical cases (Figure 7) using the within‐ and between‐subject variation in the study population, as described elsewhere [31, 39]. Outliers were counted and plotted by time point (Figure 7). As expected, the number of outliers increased at the three time points where augmented *ALAS2* levels also were detected (Figure 3), with a peak of 18/23 outliers (sensitivity of ~78%) 7 days after blood withdrawal. Importantly, *ALAS2* also surpassed the calculated thresholds following the ABMT with 10/23 outliers (sensitivity of ~43%) 3 days after the ABMT. When investigating *CA1*, the response was less clear, as evident from Figure 7. Following the ABMT, a peak of 6/23 (26% sensitivity) individuals surpassed the threshold. These findings are valuable, as they emphasize that despite the mean changes (Figure 2) not revealing a change in the level of a biomarker following an ABMT, the marker may still be valuable to investigate the changes in a personalized framework considering the normal variation as done here. Furthermore, the sensitivities of 78% and 43% after blood donation and ABMT, respectively, surpass the sensitivities previously reported using the official ABP software [3]. Here, sensitivities of 58% and 29% following donation and ABMT across all time points were reported, highlighting the potential of the current mRNA markers analyzed using an approach like the ABP. Importantly, when applying this framework, the maximal number of false positives was 4 at any time point for both biomarkers. It may be that the 47 individuals included here provided too narrow between‐subject limits, and that *ALAS2* and *CA1* population means and variation can provide a more specific and applicable setup. Future studies should attempt to elucidate this.

We next investigated whether the outliers detected by *CA1* were the same as those detected by *ALAS2* (Figure 9). *ALAS2* detected more ABMT true positives for both withdrawal and micro‐transfusion. Moreover, the number of false positives was similar for both biomarkers for withdrawal and micro‐transfusion. Interestingly, combined analysis of *CA1* and *ALAS2* for withdrawal resulted in a gain of seven true positives versus eight false positives, and for micro‐transfusion, no true positives versus one false positive; hence, most positive cases were detected using *ALAS2*, while the addition of *CA1* reduced the specificity of detection.

The aim of this study was to explore the use of RNA biomarkers as a complement to the ABP using RET% as a benchmark comparison. A profile of *ALAS2* and RET% data from one example volunteer highlighted the increases in both biomarkers during withdrawal and subsequently decreased after the ABMT (Figure S1A). In addition to the increase in sensitivity achieved by the complementarity of the two markers, *ALAS2* showed a response to ABMT when RET% was unaffected (Figure S1B). Hence, *ALAS2* may offer an advantage in confirming suspicious withdrawal, as indicated by a rise in RET%, by showing a greater increase. Similarly, confirming a micro‐transfusion should involve a notable decrease in *ALAS2* relative to the RET% value.

To mimic the role of experts in the Athlete Passport Management Unit, two investigators undertook a blinded investigation to determine which volunteers were in the ABMT and placebo groups, when considering all samples from a given individual. Among the 47 volunteers analyzed, two errors occurred: one false positive and one false negative. These findings demonstrate that an individualized analysis is important to maximize detection and reduce false positives when using the mRNA biomarkers as a complement to the ABP.

## 5 Limitations

This study has several limitations. We focused on sampling DBS at restricted and precise intervals, which is challenging to implement in the real world of doping detection due to feasibility, logistics, and cost constraints. Nonetheless, our findings demonstrate trends in mRNA biomarker levels over a high‐resolution time series. Furthermore, the detection of blood withdrawal was more pronounced because the study design involved withdrawal of 450 mL whole blood, while only ~130 mL packed red blood cells were reinfused. This evidently causes larger alterations following the blood donation in comparison with the ABMT. A limitation also discussed previously [3] was that the micro‐transfusion occurred 28 days after blood withdrawal due to logistical reasons. However, as the full hematologic recovery often occurs after 36 days, this may have influenced the micro‐transfusion results [3]. Finally, in the present evaluation of *ALAS2* and *CA1*, a detection model like the ABP was created using a purpose‐built spreadsheet and the available cohort in the present study. Thus, if implementing these biomarkers into the ABP using a different model, the results may vary somewhat. A blinded interpreter analysis was not conducted for RET%; however, RET% was available in the blinded analysis, thus complicating direct comparisons with the current benchmark variables of the ABP.

## 6 Conclusion

To conclude, the aim of this study was to determine the effect of micro‐transfusion on the biomarkers, *ALAS2* and *CA1*, using DBS samples. Following an ABMT, no relevant changes in mean levels of *ALAS2* or *CA1* were observed. However, an individualized analysis revealed that the changes in both biomarkers are similar or more pronounced than the currently used RET%. By using an ABP‐like approach, it was possible to detect individual outliers following the ABMT. Importantly, the present study indicates that *ALAS2* may be more valuable as a marker for ABMT than *CA1*, and the peak *ALAS2* sensitivity of ~48% also surpasses the official ABP software's sensitivity as reported elsewhere [3]. However, *CA1* remains a crucial marker for detecting rHuEPO doping [31]. Previously, testing of routine samples highlighted the feasibility of implementing this technique into the process of analyzing EDTA blood samples [33]. Thus, combining *ALAS2* and *CA1* with RET%, or even integrating them into the ABP as secondary markers, could further aid the indirect detection of blood doping.

## Conflicts of Interest

The authors declare no conflicts of interest.

## Supporting information


**Figure S1:** Comparison of *ALAS2* (■) and RET% (○) in two ABMT volunteers. (A) Female volunteer. (B) Male volunteer. Data are presented as percentages of baseline values. Data are presented as percentages of baseline values (y‐axis). “B” indicates the mean of baseline sample values (two baseline samples, *n* = 24; eight baseline samples *n* = 47), “3D–28D” indicates 3–28 days after blood withdrawal, “3H, 6H, and 24H" indicates “3, 6, and 24 H" after micro‐transfusion, and “2D, 3D, 6D” indicate 2, 3, and 6 days after micro‐transfusion, respectively.

**Table S1:** Total of Individual outliers in the autologous blood micro‐transfusion regarding *ALAS2* (A) and *CA1* (B).

**Data S1:** Supporting Information.

## Data Availability

The data that support the findings of this study are available in the supplementary material of this article.
