# Supplementary figures and images for: mRNA Biomarkers in Dried Blood Spots May Improve Detection of Autologous Blood Micro‐Transfusions Using an Individualized Approach
Source: Drug Test Anal. 2025 Aug 9;17(11):2291–300. doi: 10.1002/dta.3939 (PMC12580161; doi:10.1002/dta.3939)

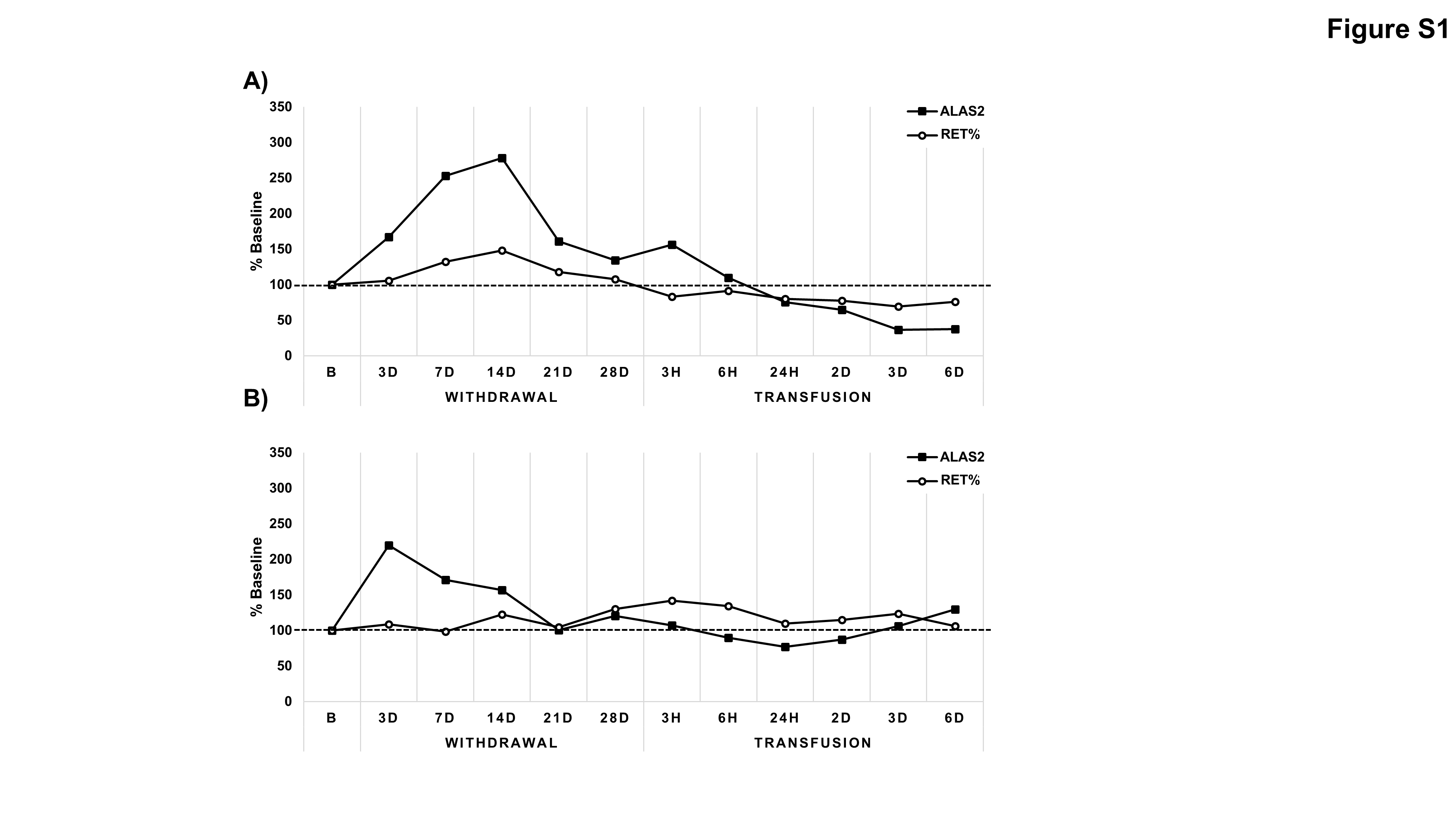

Supplement: Supplementary file 1 — Figure S1: Comparison of ALAS2 (■) and RET% (○) in two ABMT volunteers. (A) Female volunteer. (B) Male volunteer. Data are presented as percentages of baseline values. Data are presented as percentages of baseline values (y‐axis). “B” indicates the mean of baseline sample values (two baseline samples, n = 24; eight baseline samples n = 47), “3D–28D” indicates 3–28 days after blood withdrawal, “3H, 6H, and 24H" indicates “3, 6, and 24 H" after micro‐transfusion, and “2D, 3D, 6D” indicate 2, 3, and 6 days after micro‐transfusion, respectively. [file DTA-17-2291-s001.tif]
